# Surgical and survival outcomes of neoadjuvant IMRT-based chemoradiotherapy versus upfront surgery in borderline resectable pancreatic cancer: a retrospective cohort study

**DOI:** 10.3389/fonc.2026.1744117

**Published:** 2026-02-03

**Authors:** Xinru Huang, Hui Yang, Wentao Cai

**Affiliations:** 1 Burn and Wound Center, The First Affiliated Hospital of Wenzhou Medical University, Wenzhou, China; 2 Department of Anesthesiology, The First Affiliated Hospital of Wenzhou Medical University, Wenzhou, China; 3 Trauma and Emergency Surgery, The First Affiliated Hospital of Wenzhou Medical University, Wenzhou, China

**Keywords:** borderline resectable pancreatic cancer, gemcitabine, intensity-modulated radiation therapy, nab-paclitaxel, neoadjuvant therapy, R0 resection

## Abstract

**Background:**

Borderline resectable pancreatic cancer (BRPC) poses significant surgical challenges due to tumor-vessel involvement and high risk of positive margins and early recurrence. While neoadjuvant chemoradiotherapy has demonstrated potential benefits in this setting, the role of intensity-modulated radiation therapy (IMRT) combined with gemcitabine and nab-paclitaxel has not been specifically evaluated.

**Methods:**

In this single-center retrospective cohort study, we analyzed patients with histologically confirmed borderline resectable pancreatic ductal adenocarcinoma treated between 2019 and 2022 who ultimately underwent curative-intent resection. Patients either underwent upfront surgery or received neoadjuvant chemoradiotherapy consisting of gemcitabine (1000 mg/m²) and nab-paclitaxel (125 mg/m²) combined with IMRT (36 Gy in 20 fractions), followed by surgery when feasible. Overall survival (OS) and recurrence-free survival (RFS) were calculated from the date of surgery. To address baseline imbalances, propensity score overlap weighting was performed to estimate the average treatment effect in the overlap population (ATO).

**Results:**

A total of 152 patients were included, with 109 in the upfront surgery group and 43 in the chemoradiotherapy group. In unweighted analyses, median RFS was 27 months (95% CI 20.4-33.6) in the chemoradiotherapy group versus 13 months (95% CI 8.2-17.8) in the upfront surgery group (HR 0.61, 95% CI 0.39-0.94; p=0.026), and median OS was 33 months (95% CI 19.5-46.5) versus 21 months (95% CI 14.1-28.0) (HR 0.58, 95% CI 0.36-0.94; p=0.027). In ATO-weighted analyses, median RFS was 25 months (95% CI 14-not reached) versus 11 months (95% CI 8-17) (HR 0.56, 95% CI 0.35-0.88; p=0.013), and median OS was 33 months (95% CI 19-not reached) versus 17 months (95% CI 14-24) (HR 0.56, 95% CI 0.34-0.94; p=0.027).

**Conclusion:**

Neoadjuvant chemoradiotherapy with IMRT plus gemcitabine and nab-paclitaxel was associated with improved surgical and survival outcomes in patients with BRPC compared to upfront surgery. These findings support the integration of modern chemoradiotherapy into the neoadjuvant treatment paradigm for BRPC and warrant prospective validation.

## Introduction

Pancreatic ductal adenocarcinoma (PDAC) remains one of the most lethal malignancies, with the majority of patients presenting at an advanced stage and a 5-year survival rate in the single digits (1, 2). Among those patients considered for curative-intent surgery, the subgroup defined as borderline resectable pancreatic cancer (BRPC) poses unique clinical challenges (3, 4). BRPC is characterized by tumor involvement of adjacent major vessels to an extent that precludes a straightforward resection while still offering the possibility of surgical clearance with vascular resection or reconstruction (5). Patients with BRPC often experience high rates of positive resection margins (R1) and early recurrence when taken directly to surgery due to the close vascular involvement and likely presence of micrometastatic disease at diagnosis (6, 7). These challenges have prompted the exploration of neoadjuvant treatment strategies in an effort to improve outcomes for this subset.

Neoadjuvant therapy has a strong rationale in BRPC. Treating the tumor with systemic chemotherapy, with or without radiation, before surgery can potentially downstage the primary tumor, increasing the likelihood of an R0 resection (complete tumor removal with negative margins) (8). In addition, delivering therapy upfront addresses occult metastatic disease earlier in the treatment course, which may improve overall survival and helps select patients with more favorable tumor biology for surgery (9). Unlike upfront surgery, a neoadjuvant approach can spare patients with rapidly progressive disease from an ineffective operation, thereby optimizing patient selection. Given these potential benefits, there has been growing interest in neoadjuvant chemotherapy and chemoradiotherapy for BRPC, as reflected in recent clinical guidelines that recommend preoperative treatment for this group (10).

Intensity-modulated radiotherapy (IMRT) is an advanced form of radiation delivery that allows for more precise targeting of the tumor while sparing surrounding normal tissues, potentially enabling higher effective doses to the tumor or reduced toxicity (11). In pancreatic cancer, the use of IMRT could improve local tumor control by safely escalating radiation dose or by minimizing radiation-induced injury to organs such as the duodenum and stomach, thereby allowing patients to better tolerate combined modality therapy (12). Concurrently, the chemotherapy doublet of gemcitabine and nab-paclitaxel has demonstrated substantial systemic activity in pancreatic cancer and is an established first-line regimen in the metastatic setting (13). This combination has been explored in the neoadjuvant context as well, given its synergistic effect and tolerable safety profile (14).

To our knowledge, evidence specifically evaluating an IMRT-based neoadjuvant chemoradiotherapy approach combined with gemcitabine and nab-paclitaxel in BRPC remains limited. We therefore compared surgical and survival outcomes between patients treated with this neoadjuvant strategy and those undergoing upfront surgery.

## Methods

### Study design and patient selection

This was a single-center, retrospective cohort study conducted at the First Affiliated Hospital of Wenzhou Medical University, including patients diagnosed with BRPC between 2019 and 2022. BRPC was defined according to the National Comprehensive Cancer Network (NCCN) criteria based on cross-sectional imaging (10). Resectability was determined by the degree of tumor-vessel interface involving the superior mesenteric vein/portal vein (SMV/PV), SMA, common hepatic artery (CHA), and celiac axis, and baseline imaging was reviewed in a multidisciplinary setting by pancreatic surgeons and radiologists.

Inclusion criteria were: (1) age ≥18 years; (2) histologically or cytologically confirmed PDAC; (3) borderline resectable status at initial diagnosis; (4) management with one of two initial strategies, either upfront surgery or neoadjuvant chemoradiotherapy with gemcitabine plus nab-paclitaxel combined with IMRT; (5) curative-intent pancreatic resection with available imaging, operative, and pathologic data; and (6) no evidence of distant metastasis at baseline. Exclusion criteria included: prior pancreatic surgery or abdominal radiotherapy, synchronous malignancies, or incomplete clinical data. This study was approved by the ethics committee of the First Affiliated Hospital of Wenzhou Medical University, and the requirement for informed consent was waived due to the retrospective nature.

### Neoadjuvant chemotherapy and radiotherapy

Patients in the chemoradiotherapy group received a standard neoadjuvant chemotherapy regimen consisting of gemcitabine (1000 mg/m²) and nab-paclitaxel (125 mg/m²) administered intravenously on days 1, 8, and 15 of each 28-day cycle, for a total of 2 to 4 cycles depending on tolerance and response. This regimen has demonstrated favorable efficacy and tolerability in patients with advanced and borderline resectable pancreatic cancer (13, 15).

In the chemoradiotherapy group, IMRT was delivered during or after induction chemotherapy, according to institutional practice. All patients underwent CT simulation in the supine position with immobilization using a customized vacuum cushion. Simulation was performed using contrast-enhanced, pancreas-protocol CT when feasible with a slice thickness of 2–3 mm. Respiratory motion was managed with four-dimensional CT (4D-CT) and an internal target volume (ITV) approach, and daily cone-beam CT was used for image guidance. Target delineation followed institutional standards for pancreatic cancer. The gross tumor volume (GTV) included the primary pancreatic tumor and any radiographically involved regional lymph nodes. The clinical target volume (CTV) encompassed the GTV with inclusion of adjacent high-risk peripancreatic regions, excluding uninvolved organs at risk as anatomically appropriate. The ITV was generated from the 4D-CT to account for respiratory motion. The planning target volume (PTV) was generated by expanding the ITV by 5 mm in the axial direction and 10 mm in the cranio-caudal direction. IMRT plans were generated using 6–10 MV photons, and the prescribed dose was 36 Gy delivered in 20 fractions to the PTV. Plan normalization aimed for at least 95% of the PTV to receive the prescription dose, with hotspots limited to ≤107% of the prescription dose. Organs at risk (OARs), including liver, kidneys, stomach, small bowel, and spinal cord, were contoured and constrained according to the QUANTEC recommendations (16).

Postoperatively, both groups were managed according to the same institutional pathway and were intended to receive adjuvant gemcitabine when clinically eligible.

### Surgical resection and pathology evaluation

Following neoadjuvant therapy, patients were re-evaluated with contrast-enhanced CT or MRI. Those without progression were considered for surgical exploration. Pancreaticoduodenectomy, distal pancreatectomy, or total pancreatectomy was performed depending on tumor location. Vascular resection and reconstruction were conducted as needed.

Pathologic evaluation included assessment of the resection margin, lymph node status (ypN), tumor size, and tumor regression grade. R0 resection was defined as the absence of tumor cells within 1 mm of any resection margin (17, 18). Pathologic response to neoadjuvant therapy was assessed on the resection specimen by estimating the percentage of residual viable tumor cells in the treated tumor bed, in line with commonly used pathology response frameworks for pancreatic cancer. Pathologic complete response (pCR) was defined as the absence of residual viable tumor cells in the resected primary tumor bed. Major pathologic response (MPR) was defined as ≤10% residual viable tumor cells in the resected primary tumor bed (19). All resection specimens were reviewed by two gastrointestinal pathologists who assessed pathologic response and margin status independently using predefined criteria. The pathologists were not involved in clinical care; however, given the nature of the specimens, complete blinding to treatment strategy was not always feasible. Discrepancies were resolved by joint review to reach consensus.

### Outcomes and follow-up

The primary outcome was overall survival (OS), defined as the time from the date of surgery to death from any cause. Recurrence-free survival (RFS) was defined as the time from the date of surgery to the first documented recurrence (radiographic and/or histologic) or death, whichever occurred first. Patients without an event were censored at the date of last follow-up. Secondary outcomes included R0 resection rate, pCR, and MPR. Pre- and post-treatment serum CA19–9 levels were also recorded.

Patients were followed postoperatively at regular intervals: every 3 months during the first two years, every 6 months for years 3 to 5, and annually thereafter. Each follow-up included physical examination, laboratory testing (including CA19-9), and imaging studies (CT or MRI). Recurrence was defined radiographically and/or histologically. Vital status was determined from clinical records and telephone follow-up. Postoperative complications were assessed within 30 days after surgery and graded using the Clavien-Dindo classification. Major complications were defined as Clavien-Dindo grade III or higher.

### Statistical analysis

Continuous variables were summarized as mean with standard deviation or median with interquartile range, and compared using the Student’s t test or the Mann-Whitney U test, as appropriate. Categorical variables were compared using the chi-square test or Fisher’s exact test. OS and RFS were estimated using the Kaplan-Meier method. In addition to unadjusted Kaplan-Meier estimates and log-rank tests, an overlap weighting approach (average treatment effect in the overlap population [ATO]) based on the propensity score was used to balance baseline characteristics between groups and estimate the average treatment effect in the overlap population. Propensity scores were estimated using multivariable logistic regression including prespecified baseline covariates, and overlap weights were defined as 1 minus the propensity score for the chemoradiotherapy group and the propensity score for the upfront surgery group. Covariate balance before and after weighting was assessed using standardized mean differences (SMDs). Weighted Kaplan-Meier curves were constructed using overlap weights, and weighted Cox proportional hazards models with robust variance estimation were fitted to obtain hazard ratios with 95% confidence intervals. Univariate and multivariate Cox proportional hazards models were also used to identify independent predictors of OS and RFS. A two-sided p value less than 0.05 was considered statistically significant. All analyses were performed using R (version 4.5.0).

## Results

### Patient demographics and baseline characteristics

**Figure 1:**
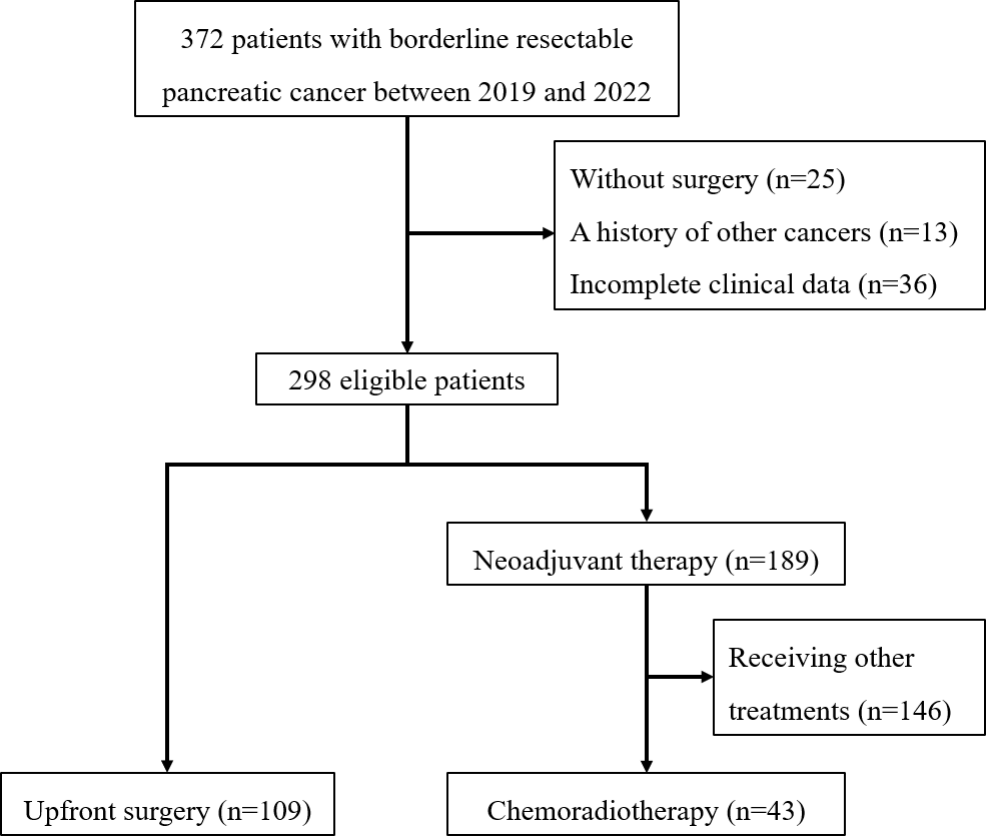
Study flowchart.

As illustrated in **Figure 1a** total of 298 patients were screened. After excluding 146 patients who received neoadjuvant treatment regimens outside the study strategy (e.g., chemotherapy alone), 152 patients who underwent curative-intent resection were included in the final analysis. Of these, 109 patients underwent upfront surgery, and 43 patients received neoadjuvant chemoradiotherapy followed by surgery.

**Table 1** summarizes the baseline characteristics of both groups. The two cohorts were comparable in terms of mean age (55 ± 10 *vs*. 53 ± 12 years, p = 0.385), sex (18.6% *vs*. 16.5% female, p = 0.758), and ECOG performance status (79.1% *vs*. 76.1% with ECOG 0, p = 0.700). A greater proportion of patients in the chemoradiotherapy group had albumin levels >35 g/L (88.4% *vs*. 79.8%, p = 0.214) and CA19–9 levels >100 U/mL (74.4% *vs*. 58.7%, p = 0.071), though these differences were not statistically significant. Tumor location was similarly distributed between the two groups (p = 0.943). However, a significant difference in vascular invasion patterns was observed (p < 0.001): patients in the chemoradiotherapy group more commonly had SMA involvement (32.6% *vs*. 8.3%), while PV involvement was more frequent in the upfront surgery group (63.3% *vs*. 44.2%). The median tumor diameter was slightly smaller in the chemoradiotherapy group (21 mm *vs*. 23 mm, p = 0.199). Baseline balance before and after ATO is shown in **Supplementary Table 1**.

**Table 1:** Patient demographics and baseline characteristics.

Characteristic	Group		p value
	Chemoradiotherapy, N = 43	Upfront surgery, N = 109	
Age, year, Mean ± SD	55 ± 10	53 ± 12	0.385
Sex, n (%)			0.758
Female	8 (18.6%)	18 (16.5%)	
Male	35 (81.4%)	91 (83.5%)	
ECOG performance status, n (%)			0.700
0	34 (79.1%)	83 (76.1%)	
1	9 (20.9%)	26 (23.9%)	
Albumin, g/L, n (%)			0.214
< 35	5 (11.6%)	22 (20.2%)	
≥ 35	38 (88.4%)	87 (79.8%)	
CA19-9, U/mL, n (%)			0.071
< 100	11 (25.6%)	45 (41.3%)	
≥ 100	32 (74.4%)	64 (58.7%)	
Tumor location, n (%)			0.943
Body, tail	20 (46.5%)	50 (45.9%)	
Head	23 (53.5%)	59 (54.1%)	
Vascular invasion type, n (%)			<0.001
Portal vein	19 (44.2%)	69 (63.3%)	
Common hepatic artery	10 (23.3%)	31 (28.4%)	
Superior mesenteric artery	14 (32.6%)	9 (8.3%)	
Tumor diameter, mm, Median (IQR)	21 (18, 27)	23 (19, 28)	0.199

### Surgical and pathologic outcomes

Among patients in the chemoradiotherapy group, 2 patients (4.7%) achieved a pCR, and 11 patients (25.6%) achieved a MPR. The R0 resection rate was significantly higher in the chemoradiotherapy group compared to the upfront surgery group (69.8% *vs*. 39.4%, respectively). Detailed surgical and pathologic results are presented in **Table 2**. Major postoperative complications (Clavien-Dindo grade III or higher) occurred in 17 of 43 patients (39.5%) in the chemoradiotherapy group and 35 of 109 patients (32.1%) in the upfront surgery group (p = 0.449).

**Table 2:** Perioperative, postoperative, and pathologic outcomes.

Outcome	Chemoradiotherapy (n=43)	Upfront surgery (n=109)	p value
Resection Margin Status			0.003
R0	30 (69.8%)	43 (39.4%)	
R1	9 (20.9%)	50 (45.9%)	
R2	4 (9.3%)	16 (14.7%)	
Pathologic Complete Response	2 (4.7%)	Not applicable	
Major Pathologic Response	11 (25.6%)	Not applicable	
Major complication (Clavien-Dindo ≥III)	17 (39.5%)	35 (32.1%)	0.449

### Survival outcomes

**Figure 2:**
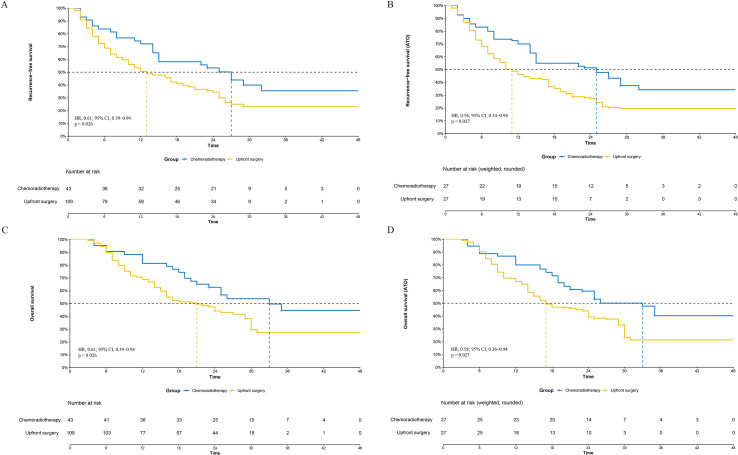
Kaplan-Meier estimated recurrence-free survival (RFS) and overall survival (OS) according to treatment before and after propensity score overlap weighting. **(A)** Unweighted RFS. **(B)** ATO-weighted RFS. **(C)** Unweighted OS. **(D)** ATO-weighted OS. ATO, average treatment effect in the overlap population; CI, confidence interval; HR, hazard ratio.

The median follow-up duration for the entire cohort was 21.5 months (range, 3-44), with a median of 20 months for the upfront surgery group and 24 months for the chemoradiotherapy group. During follow-up, 105 recurrence-free survival (RFS) events occurred (80 in the surgery group and 25 in the chemoradiotherapy group). In the unweighted analysis, the median RFS was longer in the chemoradiotherapy group (27 months, 95% CI, 20.4-33.6) than in the upfront surgery group (13 months, 95% CI, 8.2-17.8). The 1-, 2-, and 3-year RFS rates were 72.1%, 53.3%, and 35.5% in the chemoradiotherapy group, compared with 50.5%, 34.5%, and 23.1% in the upfront surgery group (HR, 0.61; 95% CI, 0.39-0.94; p = 0.026; **Figure 2A**).

In the ATO-weighted analysis, the separation of the RFS curves persisted. The ATO-weighted 1-, 2-, and 3-year RFS rates were 70.0%, 51.4%, and 34.2% in the chemoradiotherapy group and 46.0%, 27.3%, and 19.6% in the upfront surgery group. The corresponding weighted hazard ratio continued to favor chemoradiotherapy (HR, 0.56; 95% CI, 0.35-0.88; p = 0.013). The ATO-weighted median RFS was 25 months in the chemoradiotherapy group (95% CI, 14-not reached) and 11 months in the upfront surgery group (95% CI, 8-17; **Figure 2B**).

In total, 90 OS events were recorded (69 in the surgery group and 21 in the chemoradiotherapy group). In the unweighted analysis, median OS was 33 months (95% CI, 19.5-46.5) in the chemoradiotherapy group and 21 months (95% CI, 14.1-28.0) in the upfront surgery group. The 1-, 2-, and 3-year OS rates were 81.4%, 62.7%, and 44.7% in the chemoradiotherapy group and 68.8%, 44.2%, and 27.3% in the upfront surgery group (HR, 0.58; 95% CI, 0.36-0.94; p = 0.027; **Figure 2C**).

Consistent results were observed after ATO weighting. The ATO-weighted 1-, 2-, and 3-year OS rates were 79.9%, 59.5%, and 40.2% in the chemoradiotherapy group and 67.0%, 39.3%, and 21.4% in the upfront surgery group. The weighted hazard ratio remained similar in magnitude (HR, 0.56; 95% CI, 0.34-0.94; p = 0.027). The ATO-weighted median OS was 33 months in the chemoradiotherapy group (95% CI, 19-not reached) and 17 months in the upfront surgery group (95% CI, 14-24; **Figure 2D**).

### Prognostic factors

Univariate and multivariate Cox regression analyses were conducted to identify independent prognostic factors for both RFS and OS (**Table 3**, **Supplementary Table 2**). Results showed that albumin ≥35 g/L, CA19-9 >100 U/mL, and treatment group were significantly associated with RFS. In addition, CA19–9 level and treatment group also remained independently associated with OS.

**Table 3:** Univariate and multivariate analysis of overall survival.

Characteristic	Univariable					Multivariable				
	N	Event	HR	95% CI	p value	N	Event	HR	95% CI	p value
Age, year										
< 60	107	64	—	—		107	64	—	—	
≥ 60	45	26	0.89	0.56, 1.40	0.607	45	26	0.91	0.57, 1.47	0.709
Sex										
Male	126	72	—	—		126	72	—	—	
Female	26	18	1.18	0.70, 1.98	0.538	26	18	1.12	0.65, 1.94	0.688
ECOG performance status										
0	117	69	—	—						
1	35	21	1.07	0.66, 1.75	0.783					
Albumin, g/L										
< 35	27	20	—	—		27	20	—	—	
≥ 35	125	70	0.54	0.33, 0.89	0.016	125	70	0.61	0.37, 1.02	0.057
CA19-9, U/mL										
< 100	56	27	—	—		56	27	—	—	
≥ 100	96	63	1.64	1.04, 2.58	0.032	96	63	1.81	1.14, 2.87	0.012
Tumor location										
Head	82	48	—	—						
Body, tail	70	42	0.99	0.66, 1.51	0.979					
Vascular invasion type										
Portal vein	88	55	—	—						
Common hepatic artery	41	21	0.75	0.45, 1.24	0.263					
Superior mesenteric artery	23	14	0.76	0.42, 1.37	0.355					
Tumor diameter, mm										
< 20	48	20	—	—		48	20	—	—	
≥ 20	104	70	1.81	1.10, 2.98	0.019	104	70	1.66	0.99, 2.76	0.053
Group										
Upfront surgery	109	69	—	—		109	69	—	—	
Chemoradiotherapy	43	21	0.58	0.35, 0.95	0.031	43	21	0.58	0.35, 0.97	0.039

## Discussion

In this study, we found that neoadjuvant chemoradiotherapy using IMRT plus gemcitabine and nab-paclitaxel was associated with improved surgical and survival outcomes compared with upfront surgery. Patients who received neoadjuvant therapy achieved a significantly higher R0 resection rate, indicating that preoperative treatment increased the likelihood of complete tumor removal with negative margins. Enhanced pathologic responses were also observed in the chemoradiotherapy group, evidenced by a proportion of patients achieving pCR or major tumor regression in the resected specimen. These improved local outcomes translated into better long-term results: the neoadjuvant cohort demonstrated prolonged RFS and OS relative to those undergoing immediate surgery.

Our findings are consistent with and add to the growing literature supporting neoadjuvant therapy in pancreatic cancer, particularly for borderline resectable disease. Prior studies, including randomized trials, have reported potential advantages of administering therapy before surgery in this setting (20). Notably, the Dutch PREOPANC trial showed that preoperative gemcitabine-based chemoradiotherapy increased R0 resection rates and was associated with a modest improvement in overall survival compared with upfront surgery (8, 21). Likewise, retrospective studies and meta-analyses have suggested that neoadjuvant treatment may improve survival outcomes in patients with BRPC, potentially in part by increasing the proportion of patients who undergo margin-negative resection (22, 23). In our study, the observed outcomes compare favorably with several earlier reports. For instance, the median overall survival in our chemoradiotherapy group was longer than that reported in PREOPANC and some historical cohorts, although cross-study comparisons should be interpreted cautiously given differences in patient selection and treatment protocols. Nevertheless, these findings may be compatible with the systemic activity of gemcitabine plus nab-paclitaxel and the use of IMRT in our regimen. In addition, complete pathologic responses have historically been uncommon in pancreatic cancer, yet we observed a notable rate of pCR/MPR with IMRT plus chemotherapy. This observation may suggest enhanced tumor response with our combined-modality approach relative to conventional chemoradiotherapy regimens used previously (24). Regarding perioperative safety, neoadjuvant chemoradiotherapy was not associated with a higher incidence of major postoperative complications in our cohort. Major complications (Clavien-Dindo grade III or higher) occurred in 39.5% of patients after chemoradiotherapy and 32.1% after upfront surgery (p = 0.449), indicating no statistically significant difference in major postoperative morbidity among patients who proceeded to resection. Collectively, the combination of a contemporary chemotherapeutic doublet with advanced radiotherapy was associated with encouraging oncologic outcomes in our cohort, supporting further evaluation of this neoadjuvant strategy in prospective studies.

These findings may inform current treatment strategies for BRPC. Our results support consideration of neoadjuvant therapy as an initial approach for borderline resectable disease, consistent with an increasing multidisciplinary preference for preoperative regimens aimed at improving the likelihood of margin-negative resection and selecting patients most likely to benefit from surgery (25, 26). In our cohort, neoadjuvant chemoradiotherapy was associated with a higher R0 resection rate and longer survival compared with upfront surgery among patients who proceeded to resection (10). Although the mechanisms cannot be established in this retrospective analysis, earlier delivery of systemic therapy and improved local disease control are plausible contributors. Our study also suggests the feasibility of incorporating IMRT into a neoadjuvant regimen with gemcitabine and nab-paclitaxel. IMRT may facilitate delivery of preoperative radiation while limiting dose to adjacent organs at risk, which could help maintain acceptable perioperative safety.

Despite the clear benefits observed, our study has several limitations that must be acknowledged. The analysis is retrospective in nature, which introduces inherent selection biases and limits the ability to draw definitive causal conclusions. In addition, because the chemoradiotherapy group in this analysis included only patients who proceeded to resection, the results may not reflect the full intention-to-treat effect of a neoadjuvant strategy and could overestimate benefit if patients who progressed before surgery were excluded. Additionally, this was a single-center study, which may limit the generalizability of the results. The follow-up duration in our cohort, while sufficient to demonstrate significant differences in recurrence and survival, remains limited; longer follow-up is necessary to assess sustained outcomes, late recurrences, and potential long-term toxicities of the neoadjuvant regimen. Finally, our study focused on a specific chemotherapy regimen and radiation technique, so these results may not directly apply to other neoadjuvant regimens (such as FOLFIRINOX or stereotactic body radiotherapy) which are also commonly used in BRPC management. These limitations underline the need for cautious interpretation of our findings and highlight areas for future research.

In conclusion, in this single-center retrospective cohort of patients with BRPC, an IMRT-based neoadjuvant chemoradiotherapy regimen with gemcitabine and nab-paclitaxel was associated with a higher R0 resection rate and longer RFS and OS than upfront surgery, with results that were consistent after adjustment using propensity score overlap weighting. These findings suggest that incorporating IMRT into a neoadjuvant chemoradiotherapy approach for BRPC is feasible. Prospective studies are warranted to confirm these observations and to compare this regimen with contemporary neoadjuvant strategies, including chemotherapy-alone approaches. Further work to refine patient selection and to identify predictive biomarkers may help identify patients most likely to benefit from neoadjuvant therapy.

## Data Availability

The raw data supporting the conclusions of this article will be made available by the authors, without undue reservation.
