# Supplementary material for: Surgical and survival outcomes of neoadjuvant IMRT-based chemoradiotherapy versus upfront surgery in borderline resectable pancreatic cancer: a retrospective cohort study
Source: Front Oncol. 2026 Feb 3;16:1744117. doi: 10.3389/fonc.2026.1744117 (PMC12909159; doi:10.3389/fonc.2026.1744117)
Supplement: Supplementary file 1 [file Table1.docx]

**Supplementary Table 1. Baseline characteristics before and after propensity score overlap weighting (ATO) in the chemoradiotherapy and upfront surgery groups**

| Characteristic | Before ATO |  |  | After ATO |  |  |
| --- | --- | --- | --- | --- | --- | --- |
|  | Chemoradiotherapy | Upfront surgery | SMD | Chemoradiotherapy | Upfront surgery | SMD |
| n | 43 | 109 |  | 26.7 | 26.7 |  |
| Age ≥ 60 years (%) | 12 (27.9) | 33 (30.3) | 0.052 | 7.5 (28.1) | 7.5 (28.1) | <0.001 |
| Sex = Male (%) | 35 (81.4) | 91 (83.5) | 0.055 | 22.1 (82.9) | 22.1 (82.9) | <0.001 |
| ECOG performance status = 1 (%) | 9 (20.9) | 26 (23.9) | 0.070 | 5.2 (19.4) | 5.2 (19.4) | <0.001 |
| Albumin ≥ 35 g/L (%) | 38 (88.4) | 87 (79.8) | 0.236 | 23.5 (88.1) | 23.5 (88.1) | <0.001 |
| CA19-9 ≥ 100 U/mL (%) | 32 (74.4) | 64 (58.7) | 0.338 | 19.0 (71.1) | 19.0 (71.1) | <0.001 |
| Tumor location = Head (%) | 23 (53.5) | 59 (54.1) | 0.013 | 13.2 (49.4) | 13.2 (49.4) | <0.001 |
| Vascular invasion type (%) |  |  | 0.636 |  |  | <0.001 |
| Common hepatic artery | 10 (23.3) | 31 (28.4) |  | 7.2 (26.8) | 7.2 (26.8) |  |
| Portal vein | 19 (44.2) | 69 (63.3) |  | 14.2 (53.4) | 14.2 (53.4) |  |
| Superior mesenteric artery | 14 (32.6) | 9 (8.3) |  | 5.3 (19.8) | 5.3 (19.8) |  |
| Tumor diameter ≥ 20 mm (%) | 27 (62.8) | 77 (70.6) | 0.167 | 17.1 (64.1) | 17.1 (64.1) | <0.001 |
