# Supplementary material for: Surgical and survival outcomes of neoadjuvant IMRT-based chemoradiotherapy versus upfront surgery in borderline resectable pancreatic cancer: a retrospective cohort study
Source: Front Oncol. 2026 Feb 3;16:1744117. doi: 10.3389/fonc.2026.1744117 (PMC12909159; doi:10.3389/fonc.2026.1744117)
Supplement: Supplementary file 2 [file Table2.docx]

Supplementary Table 2. Univariate and multivariate analysis of recurrence-free survival

| **Characteristic** | **Univariable** | | | | | **Multivariable** | | | | |
| --- | --- | --- | --- | --- | --- | --- | --- | --- | --- | --- |
|  | **N** | **Event** | **HR** | **95% CI** | **P value** | **N** | **Event** | **HR** | **95% CI** | **P value** |
| **Age, year** |  |  |  |  |  |  |  |  |  |  |
| ˂ 60 | 107 | 73 | — | — |  | 107 | 73 | — | — |  |
| ≥ 60 | 45 | 32 | 1.01 | 0.66, 1.52 | 0.978 | 45 | 32 | 0.99 | 0.65, 1.53 | 0.979 |
| **Sex** |  |  |  |  |  |  |  |  |  |  |
| Male | 126 | 86 | — | — |  | 126 | 86 | — | — |  |
| Female | 26 | 19 | 1.13 | 0.68, 1.85 | 0.640 | 26 | 19 | 1.15 | 0.67, 1.95 | 0.617 |
| **ECOG performance status** |  |  |  |  |  |  |  |  |  |  |
| 0 | 117 | 80 | — | — |  |  |  |  |  |  |
| 1 | 35 | 25 | 1.14 | 0.73, 1.79 | 0.572 |  |  |  |  |  |
| **Albumin, g/L** |  |  |  |  |  |  |  |  |  |  |
| ˂ 35 | 27 | 23 | — | — |  | 27 | 23 | — | — |  |
| ≥ 35 | 125 | 82 | 0.53 | 0.33, 0.84 | 0.008 | 125 | 82 | 0.59 | 0.37, 0.95 | 0.029 |
| **CA19-9, U/mL** |  |  |  |  |  |  |  |  |  |  |
| ˂ 100 | 56 | 33 | — | — |  | 56 | 33 | — | — |  |
| ≥ 100 | 96 | 72 | 1.62 | 1.07, 2.44 | 0.023 | 96 | 72 | 1.76 | 1.16, 2.68 | 0.008 |
| **Tumor location** |  |  |  |  |  |  |  |  |  |  |
| Head | 82 | 54 | — | — |  |  |  |  |  |  |
| Body, tail | 70 | 51 | 1.11 | 0.76, 1.63 | 0.598 |  |  |  |  |  |
| **Vascular invasion type** |  |  |  |  |  |  |  |  |  |  |
| Portal vein | 88 | 60 | — | — |  |  |  |  |  |  |
| Common hepatic artery | 41 | 28 | 0.92 | 0.59, 1.45 | 0.731 |  |  |  |  |  |
| Superior mesenteric artery | 23 | 17 | 0.94 | 0.55, 1.61 | 0.820 |  |  |  |  |  |
| **Tumor diameter, mm** |  |  |  |  |  |  |  |  |  |  |
| ˂ 20 | 48 | 30 | — | — |  | 48 | 30 | — | — |  |
| ≥ 20 | 104 | 75 | 1.32 | 0.87, 2.02 | 0.196 | 104 | 75 | 1.20 | 0.77, 1.85 | 0.423 |
| **Group** |  |  |  |  |  |  |  |  |  |  |
| Upfront surgery | 109 | 80 | — | — |  | 109 | 80 | — | — |  |
| Chemoradiotherapy | 43 | 25 | 0.61 | 0.39, 0.95 | 0.030 | 43 | 25 | 0.59 | 0.37, 0.95 | 0.030 |
